# Modeling dynamics on the dance floor with directional swarmalators

**DOI:** 10.3389/fnbeh.2025.1534371

**Published:** 2025-02-05

**Authors:** Petri Toiviainen, Joshua S. Bamford, Marc R. Thompson

**Affiliations:** ^1^Centre of Excellence in Music, Mind, Body and Brain, University of Jyväskylä, Jyväskylä, Finland; ^2^Department of Music, Art and Culture Studies, University of Jyväskylä, Jyväskylä, Finland; ^3^Social Body Lab, Centre for the Study of Social Cohesion, School of Anthropology and Museum Ethnography, University of Oxford, Oxford, United Kingdom

**Keywords:** dance and movement, interaction, complex dynamics, swarmalators, entrainment

## Abstract

Understanding collective behavior in both biological and social contexts, such as human interactions on dance floors, is a growing field of interest. Spatiotemporal dynamics of collective behavior have previously been modeled, for instance, with swarmalators, which are dynamical units that exhibit both swarming behavior and synchronization, combining spatial movement and entrainment. In our current study, we have expanded the swarmalator concept to encompass gaze direction as a representation of visual attention. We employ the newly developed directional swarmalator model for simulating the complex spatiotemporal dynamics observed on dance floors. Our model aims to reflect the complex dynamics of collective movement, as well as rhythmic synchronization and gaze alignment. It establishes a quantitative framework to dissect how individuals on dance floors self-organize and generate emergent patterns in response to both musical stimuli and visual perception of other dancers. The inclusion of gaze direction allows for the simulation of realistic scenarios on dance floors, mirroring the dynamic interplay of human movement in rhythm-driven environments. The model is initially tested against motion capture recordings of two groups dancing in a silent disco, however, it is theoretically adaptable to a variety of scenarios, including varying group sizes, adjustable degrees of auditory and visual coupling, as well as modifiable interaction ranges, making it a generic tool for exploring collective behavior in musical settings. The development of the directional swarmalator model contributes to understanding social dynamics in shared music and dance experiences.

## 1 Introduction

Humans display a wide array of coordination behaviors of varying complexity. Collaborative work, sports, music, and dance all require interpersonal coordination to perform successfully, whether coordinating through behavior matching (imitation) or through behavioral synchrony and rhythmic entrainment (Bernieri and Rosenthal, 1991). A lot of joint action research has focused on simple, dyadic interactions. These are relatively easy to study in the lab, with two participants coordinating on a task, such as rowing, drumming, tapping, or dancing (Cuijpers et al., 2015; Dotov et al., 2022). In these instances, relatively simple measures of synchrony (e.g., cross-correlation) may be used to assess the extent of coordination. However, real social interactions are often more complex than two people moving in synchrony, and may involve large groups of people, which requires more complex means of modeling social dynamics.

According to McMahon and Isik (2023), there are three social primitives to any social interaction: contingent motion, distance, and facingness. These are the basic visual features that one may observe to determine the extent to which any two or more agents are interacting, and all three have been used to measure social interactions in dance.

Previous dance research has examined each of these social primitives. For example, contingent motion, often operationalized as a form of synchrony (Hartmann et al., 2023), has been found to predict perceived similarity between the dancers (Hartmann et al., 2019). Interpersonal distance has been measured as a proxy for social affiliation on the dance floor (Bamford et al., 2023). Finally, facingness, whether measured through head or torso orientation relative to other dancers, has been used to predict perceived interaction (Hartmann et al., 2019), or as a measure of social attention (Bamford et al., 2023; Woolhouse and Lai, 2014).

Dance provides a useful platform for studying large-scale, coordination dynamics. Although there are many examples of partner dances (Kaminsky, 2020), dance is often performed in groups in many cultures (Brown, 2022). However, most of these previous studies have focused on dyadic interactions or, in some cases, very small groups with limited spatial movement. Agent based modeling is a useful way of understanding complex behavior in humans and other animals, and some existing models may be applied to studying dance as a dynamic system.

Existing models have been used to study swarming behavior in birds, bees and other organisms. Collective behavior between individuals in a swarm can produce an emergent superorganism. For instance, Okubo (1986) model can be used to simulate a flock of birds (Reynolds et al., 2022). Models such as this may simulate the translational movement dynamics of individuals within a collective, however, they do not include the oscillatory dynamics featured in dance movements.

Other models have been used to study the behavior of two or more oscillators. The Kuramoto model describes the behavior of coupled oscillators, such that when there is sufficient coupling strength synchrony spontaneously emerges (Acebrón et al., 2005). This has since been applied to a wide range of biological phenomena, such as frogs chorusing (Aihara et al., 2008) and humans clapping at a concert (Néda et al., 2000). Another is the Haken-Kelso-Bunz (HKB) model, which was developed for modeling intraindividual synchrony between limbs, but has since been extended to interpersonal synchrony, and is notable for accommodating asymmetry, i.e., antiphase synchrony is treated as a stable state in the HKB model (Kelso, 2021). One other example, the ADaptation and Anticipation Model (ADAM), aims to simulate synchrony between individuals, while also modeling the internal adaptation and anticipation processes required for sensorimotor synchronization (SMS) in humans (Van Der Steen and Keller, 2013). This makes ADAM more specific to modeling the interactions between agents with human-like SMS abilities, while the Kuromoto and HKB models are suitable for any interactions between coupled oscillators. However, all of these models are limited to oscillatory dynamics.

Social interactions on the dancefloor involve both oscillation within and between individuals, as well as movement or spatial translation across the dancefloor, and directed attention. Swarmalators provide a potential solution to incorporate the oscillatory and translational dynamics into a single model (O’Keeffe et al., 2017). Each agent within the model is both an oscillator and a member of a swarm, which enables the study of contingent motion and interagent distance, as two social primitives. However, swarmalators still neglect the “facingness” component of any social interaction between humans.

Humans do not have an infinite attentional capacity, nor do they have eyes on the back of their head. Wirth et al. (2023) highlight the importance of visual heading in collective dynamics, emphasizing that the neighbourhood of interaction in human crowds is best explained by a visual model, where interactions are governed by optical motions and the visibility of neighbors. Keller (2023) in his theoretical model of ensemble coordination proposes three abilities that are required for SMS: attention, anticipation, and adaptation. Anticipation and adaptation are built into ADAM as discussed above (Van Der Steen and Keller, 2013), however, attention has not been incorporated. Similarly, swarmalators, in their current form, assume 360° vision (O’Keeffe et al., 2017), which limits their applicability to humans interacting on a dance floor, in which the orientation of dancers is crucial to their successful coordination (Bamford et al., 2023).

The novel solution developed in this paper is to introduce a directional swarmalator model. This maintains the oscillatory and translational dynamics of typical swarmalators (O’Keeffe et al., 2017), but also includes rotational dynamics, acknowledging the role of “facingness” in a social interaction. Each agent oscillates, can move around in a defined space, and can also change the orientation of its gaze. In addition, within this model, there is an external driving oscillation to which the agents are entrained. Within the model specified below, agents will be attracted to others that oscillate with a beat aligned to their own, and attraction can happen through both moving toward a target, and rotating to face it. An agent may also become entrained to other agents in the space, but only those within its field-of-view. Consequently, directional swarmalators offer an opportunity to study all three social primitives in large groups of dancers.

This paper specifies the directional swarmalator model with its three dynamics: translation, rotation, and oscillation. It then outlines measurements for each of these dynamics.

As circles are common formations in many dance cultures worldwide (Chauvigné et al., 2019; Sachs, 1965), we developed measures of self-organization to quantify the degree of circularity within the group, as well as centroidal alignment—the extent to which all group members were oriented toward the group’s midpoint. Finally, a phase coherence measure was used to quantify phase locking between swarmalators. Results for each of these measures were compared between simulated data from the directional swarmalator model, and real-world motion capture data from a silent disco.

## 2 Directional swarmalator model

Let the swarm consist of swarmalators *s*_*j*_, *j* = 1,…,*N*. The instantaneous state of *s_j_* is defined by five state variables. These are the position **x**_*j*_ ∈ ℝ^2^, oscillation phase θ_*j*_, azimuth of gaze direction δ_*j*_, spontaneous oscillation frequency ω_*i*_, and phase of external stimulus φ_*j*_.

For future purposes, we define the *proximity* between *s_i_* and *s_j_* as the inverse of their mutual Euclidean distance, *w*_*jk*_: = 1/|**x**_*j*_−**x**_*k*_|.

### 2.1 Translational dynamics

The translational dynamics of the model comprise three parts, those of global attraction, repulsion, and phase-and-gaze-dependent attraction. Consequently, the instantaneous velocity of *s_j_*, denoted by ${\dot{\text{x}}}_{j}$, consists of three components. First, overall attraction component constraints its distance from the origin, and is defined by


(1) $${\dot{\text{x}}}_{A\,j}=-A\,{\text{x}}_{j}\,{\left|{\text{x}}_{j}\right|}^{a-1}$$

Here *A* determines the strength of attraction and *a* defines its degree of exponential increase with distance. Second, the repulsion component prevents *s_i_* from coalescing with other swarmalators, and is defined by


(2) $${\dot{\text{x}}}_{R\,j}=-\frac{R}{N}\,\sum_{k\neq j}w_{k\,j}^{r}\,\left({\text{x}}_{k}-{\text{x}}_{j}\right)/\left|{\text{x}}_{k}-{\text{x}}_{j}\right|$$

Here *R* determines the overall strength of repulsion and the spatial decay exponent *r* dictates how the force or interaction decays with increasing distance to another swarmalators.

Third, phase-and-gaze-dependent spatial coupling is defined by


(3) $${\dot{\text{x}}}_{P\,j}=\frac{P}{N}\,\sum_{k\neq j}w_{k\,j}^{p}\,\Omega \,\left(\theta_{k}-\theta_{j}\right)\,\Upsilon \,\left(\alpha_{k\,j}-\delta_{j}\right)\,\left({\text{x}}_{k}-{\text{x}}_{j}\right)/\left|{\text{x}}_{k}-{\text{x}}_{j}\right|$$

where *P* and *p* determine the strength and spatial decay of the interaction, respectively, Ω denotes the phase coupling function, Υ the gaze coupling function, and α_*kj*_: = ∠ (**x**_*k*_ − **x**_*j*_) the azimuth angle of the vector pointing from *s_j_* to *s_k_*. The phase and gaze coupling functions should be defined so that *s_j_* is maximally attracted by *s_k_* when the two are similar in phase, and the gaze of *s_j_* is pointing toward *s_k_*. In the present instance of the model, we define the phase coupling function to be


(4) $$\Omega \,\left(\theta \right)=\frac{1+\cos\left(\theta \right)}{2}$$

Similarly, in the present instance of the model we define the gaze coupling function by


(5) $$\Upsilon \,\left(\theta \right)={\left(\frac{1+\cos\left(\theta \right)}{2}\right)}^{c}/\int_{-\pi }^{\pi }{\left(\frac{1+\cos\left(\theta \right)}{2}\right)}^{c}\,d\theta$$

**FIGURE 1:**
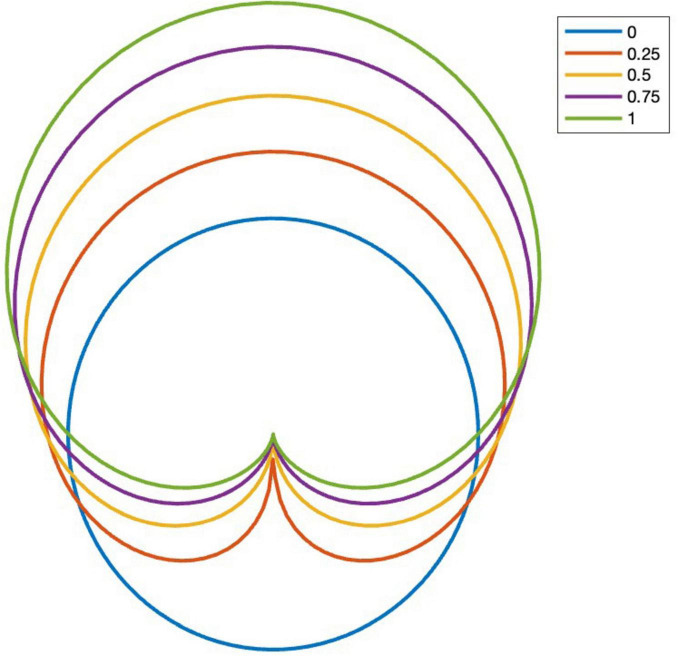
Effect of constriction parameter *c* on the gaze coupling function. Front view is on the top of this figure.

Parameter *c* affects the width of the modeled visual field and is referred to as constriction. The denominator in Eq. 5 is a normalization parameter that makes the average value of Υ independent of *c*. The constriction parameter defines the width of a swarmalator’s visual field, determining the angular region in which interactions are strongest. Higher values of narrow the visual field, making the swarmalator less sensitive to individuals outside a forward-facing region. This models the limited visual attention of real-world agents, such as dancers, who primarily interact with those within their line of sight. See Figure 1 for an example of the effect of constriction.

Finally, the total instantaneous velocity of *s_i_* is defined as the sum of the three previous terms:


(6) $${\dot{\text{x}}}_{j}={\dot{\text{x}}}_{A\,j}+{\dot{\text{x}}}_{R\,j}+{\dot{\text{x}}}_{P\,j}.$$

### 2.2 Rotational dynamics

The gaze direction of swarmalators is attracted by other swarmalators, most strongly by those that are proximal and similar in phase. Formally, the time derivative of the gaze direction of *s_j_* is defined as


(7) $${\dot{\delta }}_{j}=\frac{D}{N}\,\sum_{k\neq j}w_{k\,j}^{d}\,\Upsilon^{\prime }\,\left(\alpha_{k\,j}-\delta_{j}\right)\,\Omega \,\left(\theta_{k}-\theta_{j}\right)$$

Here *D* and *d* determine the strength and spatial decay of rotational interaction, respectively, and $\Upsilon^{\prime }\,\left(\theta \right)=\frac{d\,\Upsilon }{d\,\theta }$ . Using the gaze coupling function of Eq. (5), we get


(8) $$\Upsilon^{\prime }\,\left(\theta \right)=\frac{c\,s\,i\,n\,\Theta }{2}\,{\left(\frac{1+\cos\left(\theta \right)}{2}\right)}^{c-1}$$

### 2.3 Oscillatory dynamics

The oscillatory dynamics of *s_i_* comprises three components: spontaneous frequency, auditory entrainment to external stimulus, and visual entrainment to other swarmalators. Spontaneous frequency can, for instance, be drawn from a normal distribution centered at a mean spontaneous moving rate, ω_*j*_𝒩(μ, σ). The auditory entrainment component is expressed by


(9) $${\dot{\theta }}_{\alpha \,j}=U\,\sin\left(\varphi_{j}-\theta_{j}\right)$$

where *U* denotes the strength of auditory coupling and φ_*j*_ the phase of the external stimulus.

Visual entrainment, in turn, is expressed by


(10) $${\dot{\theta }}_{V\,j}=\frac{V}{N}\,\sum_{k\neq j}w_{k\,j}^{v}\,\Upsilon \,\left(\alpha_{k\,j}-\delta_{j}\right)\,\sin\,\left(\theta_{k}-\theta_{j}\right)$$

where *V* and *v* determine the strength and spatial decay of the interaction. According to this equation, visual entrainment is strongest to other swarmalators that are proximal and similar in phase. Finally, the time derivative of the oscillation phase is expressed as the sum of the three abovementioned terms:


(11) $${\dot{\theta }}_{j}=\omega_{j}+{\dot{\theta }}_{\alpha \,j}+{\dot{\theta }}_{V\,j}$$

## 3 Group-level measures of self-organization

Swarmalators manifest self-organization in terms of their location, direction, and oscillation phase. In the following, we propose measures that can be used to quantify the degree of self-organization as a function of time in each of these three domains. In settings where groups of swarmalators are fed with different external stimuli, such as in a silent disco, all these measures can be calculated on both global and group levels.

### 3.1 Translational self-organization

*Circularity* κ measures the degree to which the swarmalators form a circular configuration, and is operationalized as standard deviation of distances from group centroid:


(12) $$\kappa =\sigma \,\left(\left|{\text{x}}_{j}-\left\langle x\right\rangle \right|\right)$$

where $\left\langle x\right\rangle =\frac{1}{N}\,\sum_{j}{\text{x}}_{j}$ denotes the position of the group mean. The value κ = 0 indicates that the swarmalators are organized in a perfect circle.

*Grouping coefficient* ρ measures the extent to which swarmalators driven by the same stimulus are grouped together. It is operationalized as the intracluster correlation coefficient


(13) $$\rho =\frac{\sigma_{b}^{2}}{\sigma_{b}^{2}+\sigma_{w}^{2}}$$

where $\sigma_{b}^{2}$ and $\sigma_{w}^{2}$ are the between- and within-cluster variances, respectively, and ranges between 0 and 1. A cluster is defined based on the auditory stimulus received by each swarmalator, with each unique stimulus corresponding to a distinct group.

### 3.2 Rotational self-organization

*Gaze locking coefficient* γ measures the degree to which swarmalators are facing at each other. It is defined by


(14) $$\gamma =\frac{1}{N\,\left(N-1\right)}\,\sum_{k\neq j}\cos\,\left(\alpha_{k\,j}-\delta_{j}\right)$$

and ranges between −1 and 1.

*Centroidal alignment* χ measures the degree to which swarmalators are facing at the group centroid, and is defined by


(15) $$\chi =\frac{1}{N}\,\sum_{j}\cos\,\left(\varepsilon_{j}-\delta_{j}\right)$$

where ε_*j*_ denotes the azimuth angle from **x**_*j*_ to ⟨**x**⟩, ε_*j*_: = ∠(⟨**x**⟩−**x**_*j*_). Again, χ ranges between -1 and 1.

### 3.3 Oscillatory self-organization

*Phase coherence R* measures the degree of phase locking between the swarmalators, and is calculated as the norm of the Kuramoto order parameter


(16) $$R=\frac{1}{N}\,\left|\sum_{i=1}^{N}e^{i\,\theta_{i}}\right|$$

To measure local phase coherence, we first define the *individual local phase coherence* of swarmalator *j* by


(17) $$R_{j}^{\sigma }:=\frac{\left|\sum_{k}K_{j\,k}^{\sigma }\,e^{i\,\theta_{k}}\right|}{\sum_{k}K_{j\,k}^{\sigma }}$$

where


(18) $$K_{j\,k}^{\sigma }:=e^{-{\left|x_{j}-x_{k}\right|}^{2}/2\,\sigma^{2}}$$

is the spatial kernel and σ the kernel width. The index $R_{j}^{\sigma }$ thus weights the contribution of each swarmalator so that the weight decreases with increasing distance, and the value of σ determines the degree of locality in the measure. Subsequently, the *local phase coherence* *R*^σ^is calculated as the mean of $R_{j}^{\sigma }$ across all swarmalators:


(19) $$R^{\sigma }:=\frac{1}{N}\sum_{j}R_{j}^{\sigma }$$

It is straightforward to see that when σ increases, *R*^σ^ approaches the global phase synchronization measure:


(20) $$\lim_{\sigma \to \infty }R^{\sigma }=R$$

## 4 Estimating state parameters from empirical data

When the participants in a silent disco experiment have been motion-captured with, for instance, two markers on the head, achieving the position and gaze direction is straightforward. As regards the oscillation phase, it has been found in several studies that in spontaneous dance the vertical velocity of the head tends to be synchronized to the tactus-level beat of music (Toiviainen et al., 2010, Toiviainen and Carlson, 2022, Burger et al., 2014). Consequently, the oscillation phase θ_*i*_ can be estimated from the vertical velocity component of head marker, ${\dot{\text{x}}}_{i\,Z}$, by means of the analytical signal using


(21) $$\theta_{i}=∠\,\left({\dot{\text{x}}}_{i\,Z}+i\,H\,\left({\dot{\text{x}}}_{i\,Z}\right)\right)$$

where ∠ denotes the argument (direction angle in complex plane), and *H* the Hilbert transform.

## 5 Simulations

### 5.1 Silent disco experiment

A silent disco was organized in an optical motion capture lab. Twelve participants (11 females, mean age = 22.9, *SD* = 1.83) were outfitted with silent disco headsets (Silent Disco King),^1^ which had been fitted with reflective markers.

The participants were asked to move in 20 conditions while listening to either metronome sequences or excerpts of real music stimuli through the silent disco headsets, however, only two were included in the current analysis due to their relevance for testing the directional swarmalator model. The first eight conditions involved participants bouncing to auditory stimuli (metronome or music) with varying phase or frequency shifts, without any specific instructions about grouping. The next eight conditions instructed participants to form groups based on visual information while listening to the same types of stimuli. In the final four conditions, participants were asked to dance freely without specific instructions. The sequence of conditions was randomized to minimize order effects. Each condition was motion-captured using the Qualisys Oqus cameras, capturing the movements of the markers affixed to the headsets at 120°Hz.

Recruitment was conducted via advertisements to Musicology and Music Education student associations at the University of Jyväskylä, and all participants were students of the Department of Music, Arts and Culture Studies. The study complied with ethical standards, including approval from the university’s ethical review board.

**FIGURE 2:**
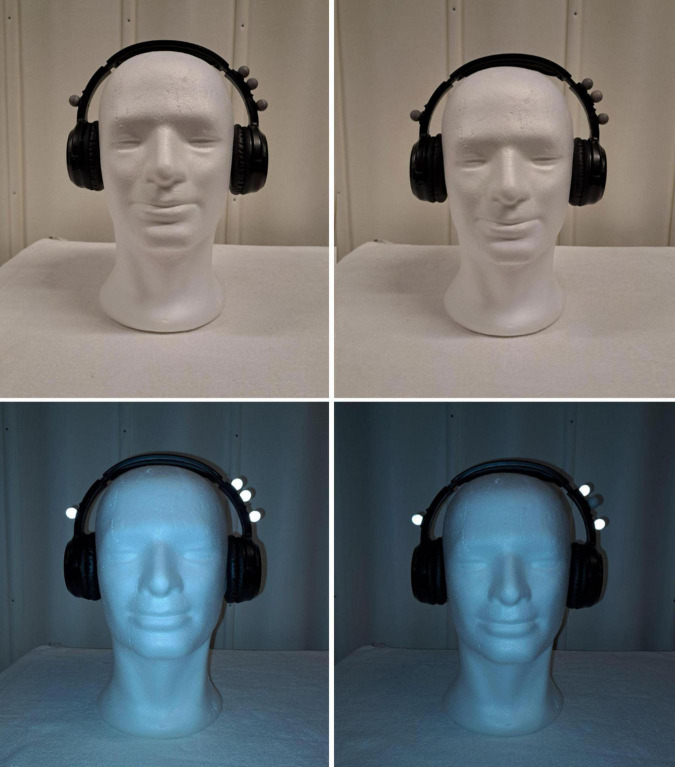
Headphones with reflective markers. Group 2 pictured on the right with the dummy marker. The top row shows the markers under normal lighting conditions, as they appeared to the participants.

In the conditions included in the present paper, the participants were randomly put into two different groups (Group 1 and Group 2). Group 1 heard the original version of the auditory stimuli, while Group 2 heard the stimuli with either a phase difference (90° or 180°) or a frequency difference (sped up) version of the stimuli. The groups were identified according to the number of markers affixed to the headsets (Group 1 headsets had three markers on the left side, while Group 2 headsets had two markers on the left side). A “dummy” marker was placed on the left side of the Group 2 markers so the participants would not be able to discern which group they were in Figure 2.

The instructions given during the conditions were either to move freely or bounce to the auditory stimuli’s main beat (tactus). Moving freely was instructed as being dance-like movements and bouncing was defined as vertical movement caused primarily by knee flexions and extensions. In some conditions participants were tasked with finding members of their groups by identifying similar or synchronous movements. Visual inspection of the motion capture data revealed that the groups swarmed more easily in the bouncing conditions than those where they were dancing.

For the sake of the current study’s modeling focus, four bouncing conditions are selected for analysis: Metronome 120 BPM with 90° and 180° phase shifts, and two musical excerpts (Girls and Boys by Blur with a 90° and Bad Romance by Lady Gaga with a 180° phase shift). The musical stimuli were time stretched to have a bpm of 120.

### 5.2 Model optimization

#### 5.2.1 Parameters to be optimized

We used the motion capture data collected in the silent disco experiment described above to perform parameter fitting for our swarmalator model. The primary goal of this fitting was to align the final configurations of the swarmalators after one minute of simulation with the observed configurations from the silent disco data as closely as possible. Due to the complexity of the model and the limited amount of empirical data available, we constrained our optimization efforts to only two parameters while maintaining fixed values for the rest.

Particularly, since the rotational dynamics represent a novel aspect of this model, we focused our optimization on parameters that directly influence this dynamic: gaze attraction strength and the constriction parameter, which affects the width of the modeled visual field. These parameters are crucial for accurately modeling how individual swarmalators adjust their gaze direction based on the positions and orientations of nearby peers, a key behavior observed in dance settings.

The fixed parameter values were adjusted to ensure that the mean distance between the swarmalators closely mirrored the trajectory observed in the empirical data from the silent disco experiment. This process involved iterative testing and refinement to achieve a dynamic alignment with real-world behavioral patterns. Consequently, we used the fixed values indicated in Table 1.

**TABLE 1:** Fixed parameter values used in the optimization.

Parameter name	Fixed value
Attraction strength	*A* = 0.1 s^–1^
Attraction range exponent	*a* = 1
Repulsion strength	*R* = 1.5 s^–1^
Repulsion decay exponent	*r* = 2
Phase-and-gaze-dependent attraction strength	*P* = 0.5 s^–1^
Spatial decay in phase-and-gaze coupling	*p* = 1
Spatial decay in rotational dynamics	*d* = 1
Auditory entrainment strength	*U* = 0.8
Visual entrainment strength	*V* = 0.4
Spatial decay in visual entrainment	*v* = 1
Attraction strength	*A* = 0.1 s^–1^

#### 5.2.2 Optimization procedure

For the parameter optimization of our swarmalator model, we utilized simulated annealing (Kirkpatrick et al., 1983), a robust optimization technique particularly suited for handling complex problems where the cost function may be non-continuous and non-differentiable. This characteristic arises in our model due to the inclusion of the grouping coefficient, which introduces discontinuities in the cost function. Simulated annealing is ideal for such scenarios as it effectively navigates the parameter landscape to find global optima, avoiding local minima that are common with more traditional gradient-based optimization methods. The optimization was implemented using MATLAB’s simulannealbnd() function.

Each of the four datasets from the silent disco experiment was used to set the initial configuration of the swarmalators, including both their positions and gaze directions. Following this initialization, we simulated the dynamics of the swarmalators for 1 min to observe the evolution of their configurations. In the simulations the phase of the external stimulus, φ_*j*_, was set to be equal to the phase of the beat of the musical stimulus the respective participant was presented with. The differential equations were numerically simulated using the Euler method with a time step of 1/120 second.

To assess the alignment between our simulated swarmalator configurations and the empirical data from the silent disco settings, we developed a composite error measure that included:

1. The spatial variance of positions, reflecting the group size,
2. The grouping coefficient, gauging the extent to which swarmalators influenced by similar stimuli grouped together,
3. The centroidal alignment, measuring the orientation of swarmalators toward the group’s centroid.

For each dataset, this error measure was calculated as the sum of the absolute differences between these three components in the empirical silent disco data and the simulated swarmalator configurations at the end of one minute. It is to be noted that for the sake of simplicity, we did not consider any measures of oscillatory self-organization in this simulation. However, with the parameter values used in the simulations, each swarmalator was accurately synchronized with its respective driving oscillation.

#### 5.2.3 Results of optimization

The optimization process identified that the parameter values for constriction (*c* = 0.252) and gaze attraction strength (*g* = 0.251) resulted in the smallest error, effectively aligning the simulated behaviors of the swarmalators with the observed dynamics at the silent disco.

**FIGURE 3:**
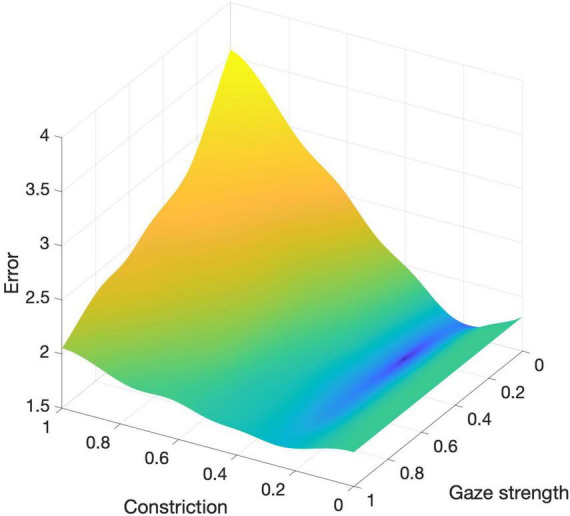
Error surface across the parameter range [0,1] for constriction (*c*) and gaze attraction strength (*g*).

Figure 3 illustrates the error surface across the parameter range [0,1] for both *c* and *g*. The visualization highlights the model’s sensitivity to changes in these parameters. Notably, the constriction parameter (*c*) has a more pronounced effect on the overall error compared to the gaze attraction strength (*g*), indicating that the width of the visual field modeled by constriction significantly impacts the accuracy of the model. Comparisons show that the model performs better with heading dynamics included (c > 0) than without (c = 0), with error values of 1.60 and 1.81, respectively.

**FIGURE 4:**
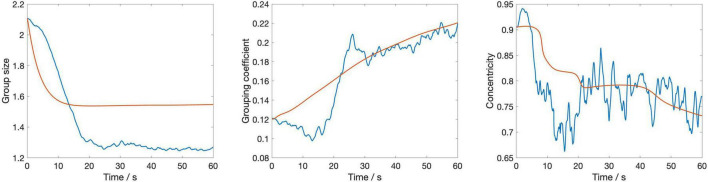
The temporal evolution of three key metrics—group size, grouping coefficient, and centroidal alignment—over a 1-min period averaged across the four stimuli. The blue lines represent empirical data from the silent disco experiment, while the red lines depict the corresponding metrics from the swarmalator model simulations.

Figure 4 displays the dynamic evolution of the three metrics used in the cost function—spatial variance, grouping coefficient, and centroidal alignment—over the first minute averaged across the four stimuli, using the optimal parameter values (*c* = 0.25 and *g* = 0.25). This visualization provides insights into how these metrics, integral to assessing the model’s performance, change over time under the influence of the identified optimal settings.

The figure shows that group size decreases sharply at the outset before stabilizing, with the model closely mirroring the empirical data but slightly underestimating the change of group size over time. The grouping coefficient begins low, indicating initial loose cohesion, and gradually increases; however, the model’s response to this increase is smoother compared to the empirical data. Centroidal alignment exhibits considerable fluctuation with an overall downward trend, suggesting a gradual reduction in central alignment, with the empirical data displaying greater variability than the model’s more uniform decline. These observations suggest that while the model captures the general trends in group behaviors effectively, its dynamics unfold slower than those observed in human interactions, highlighting a need for refining the model’s responsiveness to more accurately simulate the quick adjustments seen in real human behavior.

**FIGURE 5:**
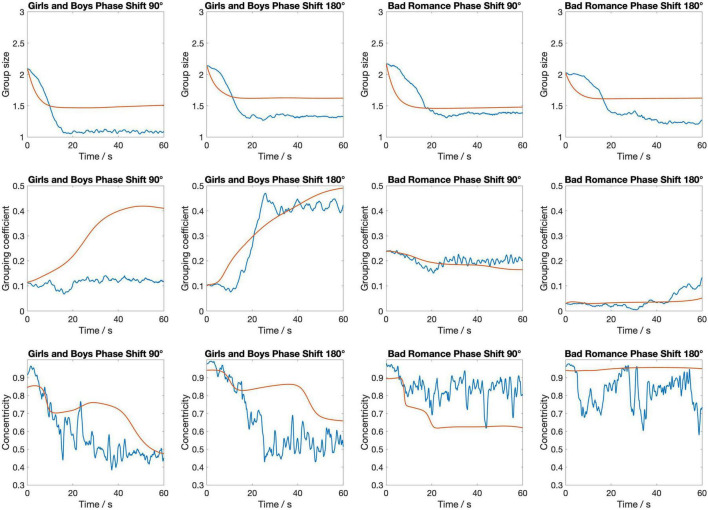
The temporal evolution of three key metrics—group size, grouping coefficient, and centroidal alignment—over a 1-min period for each of the four stimuli. The blue lines represent empirical data from the silent disco experiment, while the red lines depict the corresponding metrics from the swarmalator model simulations.

Figure 5 shows the temporal evolution of the three metrics separately for each of the four stimuli. As can be seen, there are some differences in the model’s accuracy between the stimuli. This is most notable for the second stimulus (Girls and Boys, phase shift 180°). In particular, for this stimulus the evolution of Grouping coefficient, while being of similar magnitude at the end of the 60-s interval, follows a more constant increase for the model than for the humans. Centroidal alignment for this stimulus, on the other hand, remains smoother and more stable in the model, while the human data shows greater fluctuation and a gradual decrease over time. This difference suggests that the model lacks the flexibility to capture the dynamic reorientations and variability seen in human behavior.

## 6 Discussion

The directional swarmalator model presented here may be useful in understanding how people coordinate on the social dance floor. By combining oscillatory, translational and rotational dynamics, it provides a model of group dynamics during dance. Crucially this enables the study of larger groups of dancers, going beyond dyadic interaction. In validating the model, we have also developed metrics for measuring circularity and centroidal alignment that may be useful in future research.

Through the inclusion of directionality in the swarmalator model, circular shapes tended to form between agents. Circles are common in many dance cultures around the world (Chauvigné et al., 2019; Sachs, 1965), and this may be for anatomical reasons due to the frontal placement of the human eye. In our directional swarmalator model, optimizing the gaze constriction parameter was vital. In this instance, a fairly wide gaze was found to be optimal. Previous studies have found that the horizontal field of view in humans is about 210 degrees (Strasburger, 2020), which approximates our findings within the model, although the gaze strength gradient may not perfectly reflect human data. Additionally, comparisons using our error measure indicate that the model performs slightly better when heading dynamics are included, further emphasizing the importance of gaze direction in accurately modeling collective behavior.

Although the current model approximates human behavior, there are some limitations. The most notable issue is that these directional swarmalators are too smooth in their movement. They tend to drift gradually toward an identified target, while the humans are more erratic in their motion and in their visual search behavior. This could be overcome by adding noise to the gaze direction dynamic, in order to simulate searching behavior. The directional swarmalator model is highly complex with many parameters, and the optimization of parameters was done with a very small dataset, which limits the generalizability of the model. Currently only two parameters were optimized, due to limited data availability. Collecting motion capture data with groups is time-intensive, but more data would be required for better optimization. The model could also be trained on a wider variety of data, as the silent disco task was quite limited by design. Participants were instructed to bounce, rather than dance, in order to reduce noise in the oscillatory dynamics. A more complex model may have been able to accommodate a wider variety of individual motion, beyond vertical oscillation, but that would be for future development.

The model could be further developed with a greater range of data. The silent disco task used here was restrictive in its instructions to bounce in time to the beat and to find a group. Future studies could investigate the effect of these instructions, for instance, whether participants behave differently if instructed to attend to other features of the other participants, other than their movement, or if they were instructed to sway rather than bounce, for example. The auditory stimuli could also be varied to investigate a wider variety of differences in timing or quality of movement.

In theory, the model could be extended to other behaviors beyond dance. Any situation where a group of agents form groups based upon visual features would be eligible for modeling using directional swarmalators. For instance, it could be used to study group formation dynamics for conversations at a cocktail party. Other features, other than phase matching, could be used as markers of similarity, such as types of gesture or matching clothing. Directional swarmalators may also be useful in modeling group formation in non-human animals, depending upon the importance of gaze direction. Existing swarmalator models do not account for visual fields (O’Keeffe et al., 2017). For species that move in three dimensions (e.g., schools of fish or flocks of birds) this would require adding elevation to the gaze parameter. In any case, further extensions could still be made for this model to better simulate dance movement as well. Currently swarmalators are reactive, rather than predictive, and anticipation of the beat is an important process in human sensorimotor synchronization (Keller, 2023; Van Der Steen and Keller, 2013). Adding an anticipation component to the model may increase complexity but may improve the dynamics. Overall, the directional swarmalator model presented here provides a step toward better understanding the role of visual attention on the dance floor, and potentially for other group dynamics.

## Data Availability

The raw data supporting the conclusions of this article will be made available by the authors, without undue reservation.

## Footnotes

1
https://www.silentdiscoking.com/equipment
